# Krüppel-like factor 1 (KLF1) promoted the proliferation, migration and invasion of human lens epithelial cells by enhancing the expression of Zinc Finger and BTB Domain Containing 7A (ZBTB7A) and activating Wnt/β-catenin pathway

**DOI:** 10.1080/21655979.2021.1953901

**Published:** 2021-07-24

**Authors:** Guangming Shi, Feng Yang

**Affiliations:** a Department of Ophthalmology, The People’s Hospital of Danyang; Affiliated Danyang Hospital of Nantong University, Danyang, Jiangsu Province, China; b Department of Ophthalmology, Hospital of Chengdu University of Traditional Chinese Medicine, Chengdu, China

**Keywords:** Posterior capsule opacity, cataract, KLF1, ZBTB7A, invasion

## Abstract

The epithelial–mesenchymal transition (EMT) of lens epithelial cells enhanced their proliferation and migration and therefore induced the occurrence of posterior capsule opacity (PCO). Some studies revealed that Krüppel-like factor 1 (KLF1) promoted the proliferation and invasion of multiple types of cancer cells. Besides, the expression of KLF1 was elevated in the crystalline lens of cataract patients. However, the effect of KLF1 on the development of PCO remains unclear. In this study, TGF-β2 was used for the stimulation of human lens epithelial cell line to establish EMT (SRA01/04). The KLF1 was overexpressed and knocked down in SRA01/04 cells, the proliferation, migration and invasion of which were detected by clone formation assay, wound healing and transwell assay. In addition, ZBTB7A was overexpressed in KLF1-knocked down SRA01/04 cells, the proliferation and invasion of which were also measured by clone formation assay and transwell assay. KLF1 overexpression promoted the proliferation, migration and invasion of SRA01/04 cells. Moreover, KLF1 also promoted the expression of Vimentin, snail and α-SMA in SRA01/04 cells. KLF1 enhanced the expression of ZBTB7A and β-catenin, resulting in activation of ZBTB7A and Wnt/β-catenin signaling, while overexpression of ZBTB7A abolished the inhibitory effect of knocking down KLF1 on proliferation and invasion of SRA01/04 cells. These results indicated that KLF1 promoted the proliferation, migration and invasion of human lens epithelial cells by activating ZBTB7A and Wnt/β-catenin signaling pathway.

## GRAPHICAL ABSTRACT

**Figure:**
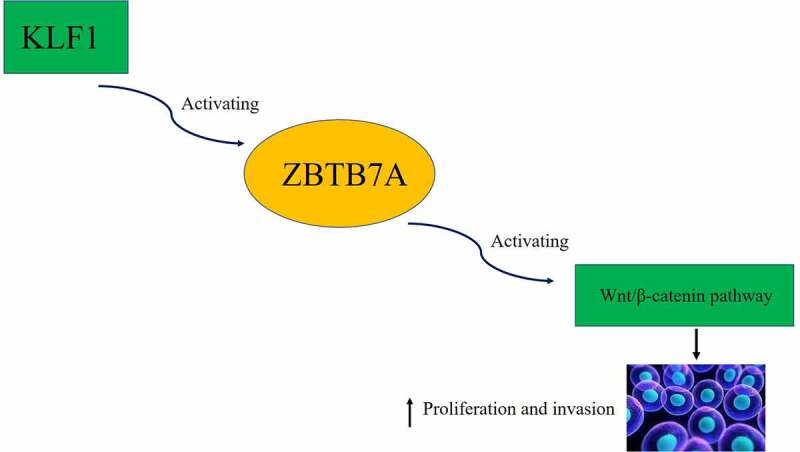

## Introduction

Lens opacity caused by cataract is one of the crucial causes of visual disturbance [1]. Severe cataracts can also cause the blindness of patients, which in turn affected the quality of life of patients. Surgery is currently the main clinical treatment for cataracts, and there is still a possibility of recurrence of cataracts after surgery. In addition, surgery will lead to a series of postoperative complications such as posterior capsule opacity (PCO) [2]. Previous study has shown that the enhanced migration and occurrence of epithelial–mesenchymal transition (EMT) of lens epithelial cells promoted the incidence of PCO [3,4]. However, the pathogenic mechanism behind PCO is still unknown. As is well known, transforming growth factor β (TGF-β) participated in modulating multiple types of cell physiological processes and the production of signal factors. Furthermore, the expression of TGF-β also induced the occurrence of EMT. Meanwhile, as a multifunctional growth factor, regulation of the expression of TGF-β also affected the invasiveness, inflammation and viability of different types of cells [5]. TGF-β2 is a member of the TGF-β family, which can modulate the migration of human lens epithelial cells (hLECs) by activating the EMT process [6]. Thus, the induction of hLECs EMT by TGF-β2 was a common PCO cell model, which was used to study the pathogenesis of PCO.

In addition, Krüppel-like factor (KLF) plays a role in the DNA transcription mechanism and participates in diverse biological processes, such as proliferation, differentiation, invasiveness and apoptosis of cells [7,8]. Krüppel-like factor 1 (KLF1) is also a member of the KLF family, and KLF1 also plays a critical role in regulating cell proliferation and migration. Moreover, KLF1 is highly expressed in a variety of tumors, such as cervical cancer. The expression of KLF1 promoted the proliferation and EMT of cervical cancer cells by activating PI3K/Akt pathway [9]. KLF1 is also associated with drug resistance of ovarian cancer cells [10]. In addition, the results of oligonucleotide microarray hybridization experiment indicated that the expression of KLF1 was statistically significantly increased in the lens of cataract patients compared to the normal lens [11]. However, the effect of KLF1 on the development of PCO remains elusive.

Furthermore, some study has found that the expression of KLF1 increased the expression of fetal globin repressor Zinc Finger and BTB Domain Containing 7A (ZBTB7A) in erythroid cells [12]. ZBTB7A is a member of the POK protein family and plays a critical role in the process of cell differentiation [13,14]. Moreover, ZBTB7A can regulate the activity of Wnt signaling pathway in non-small cell lung cancer cells and thereby accelerating the cancer progression [15]. Moreover, the activation of Wnt signaling pathway was related to the proliferation and epithelial–mesenchymal transition of human lens epithelial cells [13]. Ectopic activation of Wnt/β-catenin signaling pathway in lens fiber cells led to the occurrence of cataract and abnormal differentiation of fiber cells [16]. In addition, β-catenin modulated the TGF-β-induced EMT process of these cells in the lens by interacting with Smad3 [17]. However, whether KLF1 can affect the EMT process of lens epithelial cells by modulating the expression of ZBTB7A is unclear. Therefore, the effect of KLF1 on the proliferation and invasion of human lens epithelial cells stimulated with the TGF-β2 was investigated, which will provide a new potential therapeutic target for the treatment of PCO.

## Material and methods

### Cell culture and treatment

Human lens epithelial cell line (SRA01/04) was obtained from the ATCC (Manassas, VA, USA), which were cultured with RPMI-1640 (Hyclone, USA) medium supplemented with 10% fetal bovine serum (Gibco, USA). Cells were cultured at 37°C in the incubator with humid atmosphere supplemented with 5% CO_2_. The TGF-β2 (0 ng/ml and 10 ng/ml, Abcam, ab277760) was used for the stimulation of these cells. The lentivirus for KLF1 knockdown and overexpression and ZBTB7A overexpression were got from the Genechem (Shanghai, China). The polybrene (Genechem, Shanghai, China) was applied for enhancing the transfection efficacy of the lentivirus into SRA01/04 cells.

### Collection of clinical samples

Twenty cataract lens capsules were collected from 20 cataract patients (Age: 36–60 years old). There is no trauma to the eyes of these cataract patients. Lens were collected from patients during the cataract surgery. Twenty phacocysts collected from the volunteers (Age: 36–60 years old) who have no eye disease or damage were used as control. All the patients and volunteers consented this experiment. The experiment was checked and approved by the ethics review committee of The People’s Hospital of Danyang.

### Immunohistochemical staining

The lens capsules were embedded into paraffin and cut into sections. Next, these tissues were dewaxed with the xylene and rinsed with the PBS for three times and blocked with the BSA (Beyotime, China). Then, these sections were incubated with the primary antibody (KLF1 antibody, Abcam, ab97110) at 4°C overnight followed by an incubation with secondary antibody (Donkey Anti-Goat IgG, Abcam, ab97110) for 2 hours. Finally, sections were reacted with the TMB substrate (Millipore, USA) and observed under the electron microscope (Olympus, Japan).

### EdU staining

Cells were plated into the 96 well plates, which were incubated with the EdU for 2 hours at 37°C. Finally, these cells were stained with the DAPI (Invitrogen, USA) and observed with the fluorescence microscope (Olympus, Japan).

### Immunofluorescence

Cells were cultured on the glass slide, which were then rinsed with PBS for three times and fixed with the 4% paraformaldehyde. Next, cells were treated with 0.5% Triton X-100 (Beyotime, China) to increase the permeability of cells. After that, cells were blocked with BSA (Beyotime, China) for 2 hours and incubated with the primary antibodies at 4°C overnight. Vimentin (Abcam, ab8978), α-SMA (Abcam, ab5831) and β-catenin (Abcam, ab223075) were applied. Followingly, cells were incubated with the secondary antibodies including Goat polyclonal Secondary Antibody to Rabbit IgG (Abcam, ab150077) and Goat Anti-Mouse IgG (Abcam, ab205719) in dark room for 2 hours. Finally, cell nucleus of was stained with the DAPI (Invitrogen, USA) and the fluorescence was observed using the laser scanning confocal microscope (Olympus, Japan).

### CCK-8 assays

Cells were plated into the 96 well plates and treated with the CCK-8 (Dojindo) at 37°C for 2 hours. Finally, the absorbance of these cells was determined with the microplate reader (Thermo Fisher Scientific, USA).

### Clone formation experiment

Cells were plated into the 60 mm culture dish (600 cells per dish) and cultured for 2 weeks. Then, cells were fixed with 70% ethyl alcohol and stained with the crystal violet (Thermo Fisher Scientific, USA). The number of clones was calculated with the microscope (Olympus, Japan).

### Wound healing assays

Cells were plated into the six well plates and cultured with serum-free medium for 12 hours. Then, the scratch was created with the tweezers and photographed with the microscope (Olympus, Japan). Next, the serum-free medium was replaced with complete culture medium. After 24 hours, the scratch was photographed again and the width of the scratch was determined with the software (Image J, National Institutes of Health, USA) [18].

### Transwell assays

Before the experiments, cells were cultured with serum-free medium for 12 hours. Then, cells were cultured in matrix gel (BD, USA) diluted by serum-free medium in the upper layer of the Boyden room (8 μm, Corning, USA), while the complete medium was infused into the lower room of the Boyden room and cells were placed in the incubator for 24 hours. After that, cells on the reverse of bottom membrane were fixed with the ethanol solution and stained with the crystal violet. Finally, the number of these cells was calculated under the microscope (Olympus, Japan).

### RT-PCR

Total RNA was extracted with the Trizol (Thermo Fisher Scientific, USA). Commercial kit (Takara, Japan) was used for the reverse transcription of the RNA. Then, ABI 7500 system (Thermo Fisher Scientific, USA) was used for the amplification of the cDNA. The results were analyzed with the 2^−∆∆Ct^ method. The primers used in this research were forward primer of KLF1 5ʹ-GAAGAGGACGATGAGAGGGG-3ʹ reverse primer of KLF1 5ʹ-ATCCTCCGAACCCAAAAGC-3ʹ GAPDH forward primer 5ʹ-CCATCTTCCAGGAGCGAGAT-3ʹ GAPDH reverse primer 5ʹ-TGCTGATGATCTTGAGGCTG-3ʹ [19].

### Western blotting

Protein samples were collected with the RIPA buffer (Beyotime, China). Commercial kit (Invitrogen, USA) was used for the separation of the proteins in nucleus and cytoplasm. Then, BCA (Beyotime, China) kit was used for the determination of the concentration of proteins. Ten percent SDS PAGE gel was used for the separation of proteins. Then, proteins were transferred to the PVDF membrane (Millipore, USA) and the membranes were blocked with the BSA (Beyotime, China) followed by incubation with the primary antibodies at 4°C overnight. The primary antibodies used in this research were KLF1 (Abcam, ab175372), E-cadherin (Abcam, ab40772), Vimentin (Abcam, ab92547), α-SMA (Abcam, ab108424), Snail (Abcam, ab216347), ZBTB7A (Abcam, ab175918), c-myc (Abcam, ab32072), β-catenin (Abcam, ab32572), β-tubulin (Abcam, ab18207), Histone H3 (Abcam, ab215728) and β-actin (Abcam, ab8226) antibodies. These antibodies were diluted with the BSA at the ratio of 1:1000. Then, membranes were incubated with the secondary antibodies for 2 hours. Finally, immunoreactive signals were measured with the western blotting substrate (Millipore, USA). The results of this experiment were analyzed with the Image J.

### Statistical analysis

The GraphPad Prism 7.0 was used for the analysis of the data. The data was presented as mean ± SD (n = 3). The comparison between different groups was performed with the student’s t-test. *P* < 0.05 was considered as statistically significant difference.

## Results

In this study, the effect of KLF1 on the proliferation and invasiveness of human lens epithelial cells was investigated. We speculated that KLF1 could promote the proliferation and invasion of human lens epithelial cells by enhancing the expression of ZBTB7A. Therefore, the TGF-β2 was used for the stimulation of human lens epithelial cells and the KLF1 overexpression and knockdown was established that the influence of KLF1 overexpression or knockdown on proliferation and invasiveness of human lens epithelial cells was checked.

### Stimulation of TGF-β2 promoted the expression of KLF1 in human lens epithelial cells

**Figure 1.**
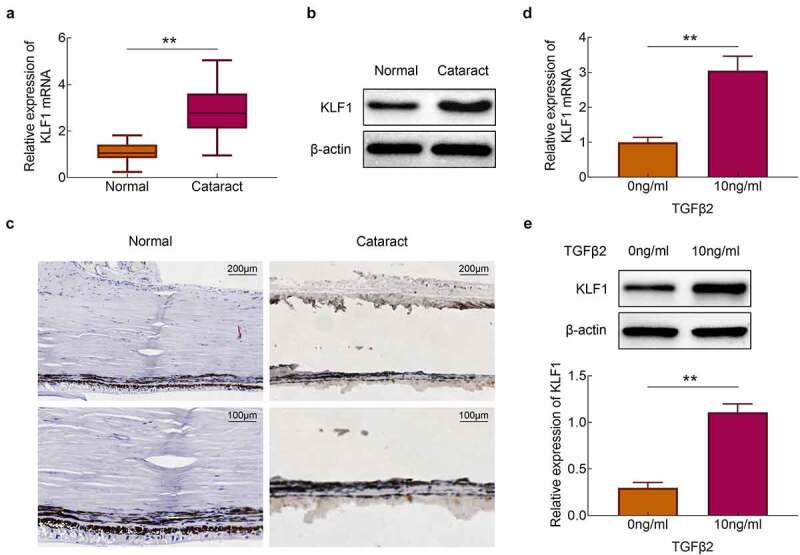
Stimulation of TGF-β2 activated the expression of KLF1 in human lens epithelial cells. (a, b) The expression of KLF1 in lens capsule tissues was detected with the RT-PCR and western blotting. (c) The expression of KLF1 in lens capsule tissues was detected by immunohistochemical staining. (d, e) The expression of KLF1 in human lens epithelial cells was detected with the RT-PCR and western blotting. * *p* < 0.05, ** *p* < 0.01, *** *p* < 0.001

The expression of KLF1 in the lens capsules of cataract patients and normal populations was detected with the RT-PCR and western blotting. Results showed that the expression of KLF1 was enhanced in the lens capsules of cataract patients compared to the normal population (Figure 1(a) and 1(b)). Similarly, the results of immunohistochemistry also showed that the expression of KLF1 in lens capsule tissues was increased after the occurrence of the cataract (Figure 1(c)). Next, TGF-β2 (10 ng/ml) was used for the stimulation of SRA01/04 cells. We found that the expression of KLF1 was enhanced in these cells by TGF-β2 stimulation (Figure 1(d,e)). These results implied that TGF-β2 stimulation promoted the expression of KLF1 in human lens epithelial cells.

### Knockdown of KLF1 suppressed the TGF-β2 induced proliferation of human lens epithelial cells

**Figure 2.**
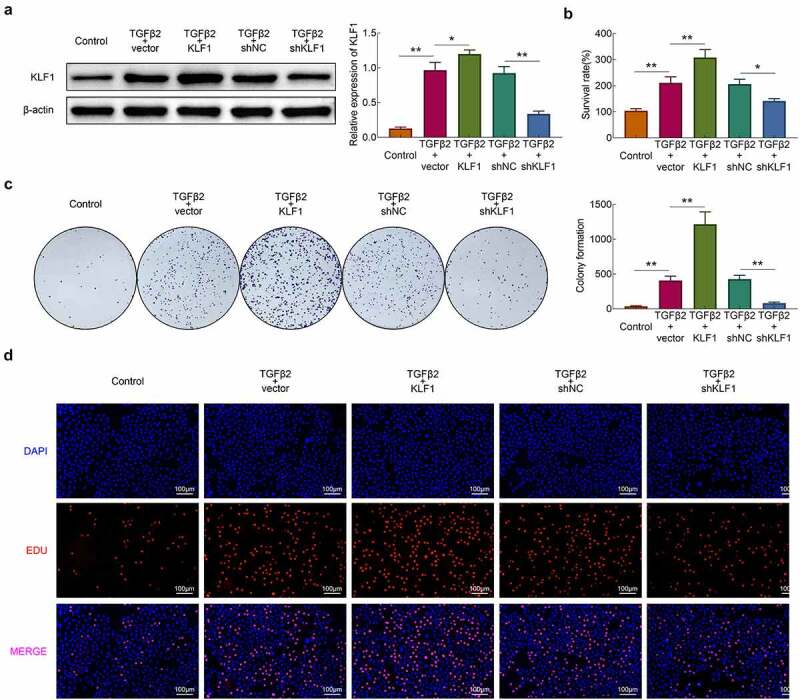
KLF1 enhanced the proliferation of human lens epithelial cells. (a) The expression of KLF1 in human lens epithelial cells determined by western blotting. (b) The survival rate of human lens epithelial cells detected by CCK-8 assay. (c) The proliferation of human lens epithelial cells detected by clone formation assay . (d) The proliferation of human lens epithelial cells determined by EdU assays. * *p* < 0.05, ** *p* < 0.01, *** *p* < 0.001

In this part, KLF1 was overexpressed and knocked down in SRA01/04 cells by plasmid transfection. The western blotting results revealed that the expression of KLF1 rised in overexpression group and declined in knockdown group. Next, TGF-β2 (10 ng/ml) was used for the stimulation of cells and CCK-8 was used for detecting the survival rate of cells. As shown in Figure 2(b), stimulation of TGF-β2 enhanced the survival rate of cells. Furthermore, overexpression of KLF1 strengthened this effect of TGF-β2 on SRA01/04 cells, while knocking down KLF1 got an inverse result. The results of clone formation assays and EdU assays also showed that overexpression of KLF1 enhanced the proliferation of SRA01/04 cells induced by TGF-β2, while which was inhibited by KLF1 knockdown (Figure 2(c,d)). These results indicated that suppression of KLF1 inhibited the TGF-β2 induced proliferation of human lens epithelial cells.

### Knockdown of KLF1 restricted the TGF-β2 induced migration and invasion of human lens epithelial cells

**Figure 3.**
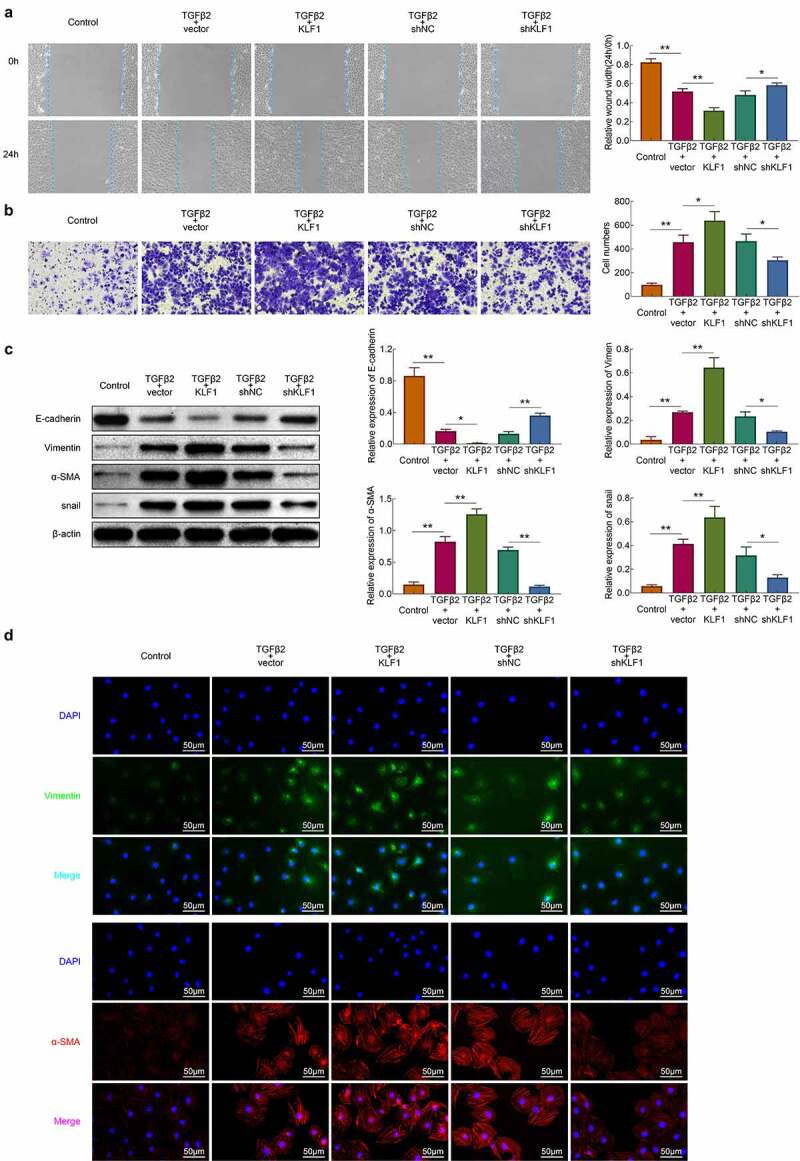
KLF1 promoted the migration and invasion of human lens epithelial cells by activating the EMT. (a) The migration of human lens epithelial cells detected by wound healing assay. (b) The invasion of human lens epithelial cells explored by transwell. (c) The expression of EMT related proteins determined by western blotting. (d) The expression of EMT associated proteins in human lens epithelial cells detected by immunofluorescence. * *p* < 0.05, ** *p* < 0.01, *** *p* < 0.001

The migration and invasion of SRA01/04 cells were detected with the wound healing and transwell assays, respectively. The stimulation of TGF-β2 enhanced the migration and invasion of SRA01/04 cells. Moreover, overexpression of KLF1 promoted this efficacy of TGF-β2 on SRA01/04 cells, while which was suppressed by KLF1 knockdown (Figure 3(a,b)). The stimulation of TGF-β2 can induce the occurrence of the EMT and promote the invasion of multiple types of cells [20,21]. Therefore, the expression of EMT related proteins was detected by western blotting and immunofluorescence. Figure 3(c,d) showed that TGF-β2 stimulation promoted the expression of Vimentin, α-SMA, snail and inhibited the expression of E-cadherin in SRA01/04 cells. In addition, overexpression of KLF1 heightened the effect of TGF-β2 on the expression of Vimentin, α-SMA and snail proteins, which was repressed by KLF1 downregulation in SRA01/04 cells. The results suggested that decreasing KLF1 alleviated the invasiveness of human lens epithelial cells induced by the TGF-β2.

### KLF1 activated the Wnt/β-catenin pathway by promoting the expression of ZBTB7A

**Figure 4.**
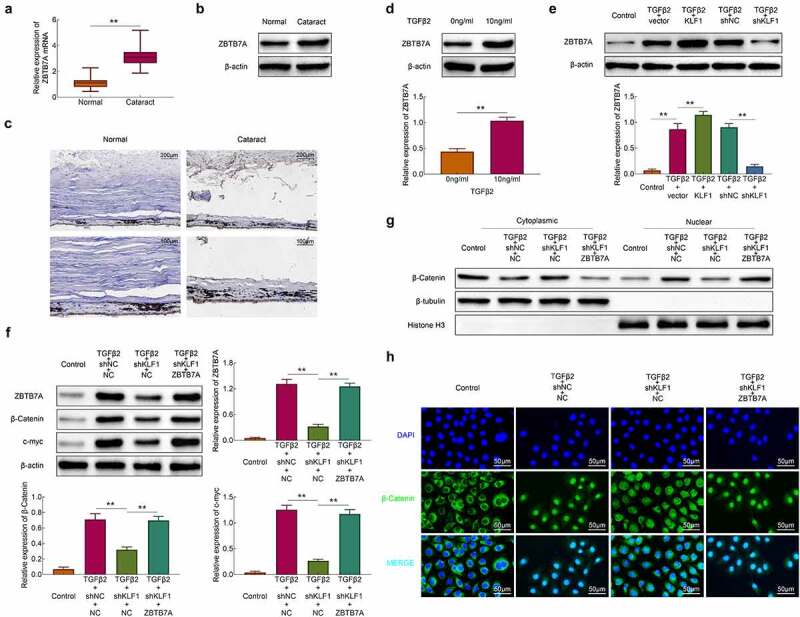
KLF1 activated the expression of ZBTB7A in human lens epithelial cells. (a, b) The expression of ZBTB7A in lens capsule tissues detected by RT-PCR and western blotting. (c) The expression of ZBTB7A in lens capsule tissues detected by immunohistochemical staining. (d, e) The expression of ZBTB7A in human lens epithelial cells detected by western blotting. (f) The expression of β-catenin, c-myc and ZBTB7A in human lens epithelial cells was determined with the western blotting. (g) The expression of β-catenin in nucleus and cytoplasm of human lens epithelial cells was determined with the western blotting. (h) The expression of β-catenin in human lens epithelial cells detected by immunofluorescence. * *p* < 0.05, ** *p* < 0.01, *** *p* < 0.001

Some study revealed that KLF1 induced the expression of ZBTB7A in erythroid cells and the higher levels of ZBTB7A activated the Wnt/β-catenin pathway [12]. Here, the expression of ZBTB7A in lens capsules of cataract patients and normal people was detected. Results in Figure 4(a,b) showed that the expression of ZBTB7A both in mRNA and protein levels in lens capsule tissues of cataract patients was higher than that in normal people. The immunohistochemistry results also showed the expression of ZBTB7A was enhanced in lens capsule tissues of cataract patients compared to the normal people (Figure 4(c)). SRA01/04 cells were stimulated with TGF-β2 and the results showed that the expression of ZBTB7A was enhanced by TGF-β2 stimulation (Figure 4(d)). It was also found that overexpression of KLF1 heightened the promotional effect of TGF-β2 on the expression of ZBTB7A in these cells, while the inhibition of KLF1 further suppressed the expression of ZBTB7A in SRA01/04 cells (Figure 4(e)). Then, the ZBTB7A was overexpressed in KLF-knocked down SRA01/04 cells and the expression of β-catenin was detected with western blot assay. As shown in Figure 4(f), knockdown of KLF1 inhibited the expression of ZBTB7A, c-myc and β-catenin, which were rescued by the overexpression of ZBTB7A in these cells (Figure 4(f)). The expression of β-catenin in nucleus and cytoplasm also showed that the transformation of β-catenin from cytoplasm to nucleus was suppressed by KLF1 downregulation, which was also reversed by ZBTB7A overexpression. overexpressing ZBTB7A also led to the accumulation of β-catenin in the nucleus of human lens epithelial cells (Figure 4(g)). The immunofluorescence results in Figure 4(h) also revealed that overexpression of ZBTB7A promoted the distribution of β-catenin in nucleus. These results indicated that KLF1 activated the Wnt/β-catenin pathway in human lens epithelial cells through promoting the expression of ZBTB7A.

### Overexpression of ZBTB7A recovered the proliferation and invasion of human lens epithelial cells

**Figure 5.**
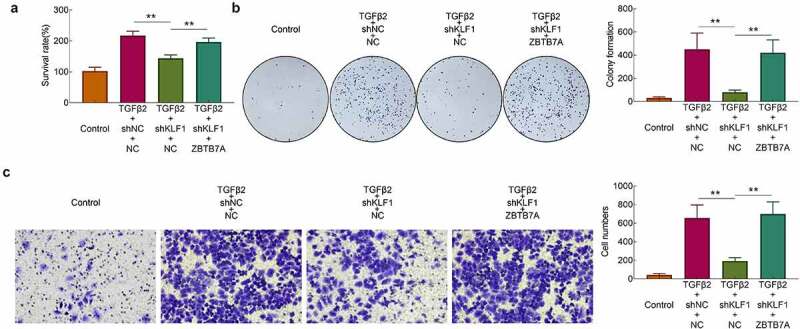
Overexpression of ZBTB7A abolished the KLF1 knockdown induced lower proliferation and invasion of human lens epithelial cells. (a) Survival rates of human lens epithelial cells determined by CCK-8 assay. (b) The proliferation of human lens epithelial cells detected by clone formation assay . (c) Invasion of human lens epithelial cells determined by transwell assay. * *p* < 0.05, ** *p* < 0.01, *** *p* < 0.001

After the overexpression of ZBTB7A, the survival rates of cells were detected with the CCK-8 assay. Results (Figure 5(a)) showed that knockdown of KLF1 induced the decrease of the survival rates in SRA01/04 cells. However, overexpression of ZBTB7A enhanced the survival rates of cells. The results of clone formation assays also revealed that overexpression of ZBTB7A abolished the inhibitory effect of knocking down KLF1 on cell proliferation (Figure 5(b)). Finally, the invasion of SRA01/04 cells was determined with the transwell experiment. It was found that knockdown of KLF1 suppressed the invasion of SRA01/04 cells, while which was rescued by the overexpression of ZBTB7A (Figure 5(c)). Results of this part implied that overexpression of ZBTB7A counteracted the inhibitory effect of KLF1 knockdown on the proliferation and invasiveness of human lens epithelial cells.

## Discussion

Cataract is one of the most critical causes of blindness and visual impairment in the world [22]. Surgery is still the main clinical treatment for cataract. However, the performance of cataract surgery could also induce the complication of cataract such as PCO. After cataract surgery, abnormal cell growth on the lens capsule often induced tissue fibrosis and secondary vision loss, which was called as PCO [23]. The occurrence of EMT of human lens epithelial cells was considered as the main cause of PCO [24]. However, the occurrence of EMT was also associated with the post-operative tissue repair of glaucoma and other eye diseases [25]. Therefore, regulating the occurrence and development of EMT is of great significance for the treatment of various eye diseases including PCO. In this study, we clarified that inhibition of KLF1 expression alleviated the proliferation and invasiveness of human lens epithelial cells by suppressing the EMT, which provides a new target for the treatment of PCO.

It has been reported that the expression of KLF1 enhanced the proliferation, migration and invasion of cervical cancer cells [9]. Moreover, some study revealed that KLF1 promoted the proliferation and invasiveness of breast cancer cells by activating the EMT process [26]. The expression of KLF1 was demonstrated to be enhanced in the lens of cataract patients [11]. Consistent with previous report, the expression of KLF1 was also promoted in the lens capsule tissues of cataract patients compared to the normal people in this study. Moreover, the expression of KLF1 promoted the proliferation, migration and invasion of human lens epithelial cells. Overexpression of KLF1 also increased the expression of EMT associated proteins (Vimentin, α-SMA and snail), which suggested that KLF1 promoted the proliferation and invasion of human lens epithelial cells by activating EMT process.

The occurrence of EMT was associated with the activation of Wnt/β-catenin pathway [27]. The activation of Wnt/β-catenin pathway can enhance the expression of EMT related proteins and induce the occurrence of EMT [28]. Furthermore, the transformation of β-catenin from cytoplasm to nucleus in human lens epithelial cells led to the occurrence of EMT and therefore induced the cataract [29]. ZBTB7A has been reported can enhance the development of non-small cell lung cancer by activating the Wnt/β-catenin pathway [15]. KLF1 was found to be able to affect the expression of ZBTB7A in erythroid cells [12]. In this research, KLF1 was also found to promote the expression of ZBTB7A in lens capsule tissues of cataract patients and human lens epithelial cells. Increasing ZBTB7A expression induced the expression of β-catenin and c-myc in cells. Moreover, overexpression of ZBTB7A abolished the inhibitory effect of KLF1 knockdown on proliferation and invasion of human lens epithelial cells. The mechanism may be that overexpression of ZBTB7A promoted the transformation of β-catenin from cytoplasm to nucleus in human lens epithelial cells, which was suppressed by knockdown of KLF1. These results indicated that inhibition of KLF1 expression suppressed the proliferation and invasion of human lens epithelial cells by inhibiting the Wnt/β-catenin pathway and EMT. However, the effect of KLF1 on the development of PCO *in vivo* was not determined, which will be explored in further study.

## Conclusion

In conclusion, the effect of KLF1 on the proliferation and invasion of human lens epithelial cells was detected and the results implied that KLF1 enhanced the proliferation, migration and invasion of human lens epithelial cells by inducing the activation of Wnt/β-catenin pathway and EMT process, which will provide a new potential therapeutic target for the clinic treatment of cataract and PCO.

## Highlight

1. Stimulation of TGF-β2 enhanced the expression of KLF1 in human lens epithelial cells.
2. KLF1 knockdown repressed the proliferation and invasion of human lens epithelial cells.
3. KLF1 activated the Wnt/β-catenin pathway in human lens epithelial cells.
4. ZBTB7A reversed the inhibitory effect of KLF1 knockdown on the invasiveness of human lens epithelial cells.
